# New paradigms, new Hopes: the need for socially responsible research on carcinogenesis

**DOI:** 10.1186/1477-3163-4-22

**Published:** 2005-11-21

**Authors:** Gopala Kovvali

**Affiliations:** 1 Gopala Kovvali Ph.D. Editor-in-Chief, Journal of Carcinogenesis, Founder President, Carcinogenesis Foundation, 22 Heritage Drive, Edison, NJ 08820, USA

It has been three years since the publication of the first article in the Journal of Carcinogenesis; it was the editorial that espoused the need for the launch of the journal. Having seen three springs and three falls, it is time to ask where we are as a journal and where we want to be in the years to come.

The past three years have been satisfying publishing years for JC, given the fact that it is an online publication with processing charges paid by the authors under the open access model and there has been stiff competition from the established print journals. The quality of manuscripts published in JC is very impressive and I am confident that it will only improve as the journal grows. Scientists seem to be conditioned to measure the quality of a journal and the articles it publishes in terms of 'impact factor'. I am glad that JC has made a significant impact on the carcinogenesis research community and I am sure that an impact factor for the journal will follow soon.

As we enter our fourth year, we have a reason to be upbeat. We have been fortunate in attracting several international scientists, both from industry and academia, to the Editorial Board. I am especially pleased that Dr. Rishab Gupta, director of immunodiagnosis and Vice-president for Education at John Wayne Cancer Institute, joined the editorial board as a managing editor. He brings outstanding leadership and vision to the editorial board. I am also pleased to announce that Carcinogenesis Foundation  was recently founded with New Jersey, USA, as a home base.

The formation of the Carcinogenesis Foundation is a historic opportunity. While its goal is to promote and advance research in the field of carcinogenesis, it has a specialized mission of understanding the phenomenon of increased cancer incidence among individuals who migrate to the western countries. The phenomenon is conceptualized and can be called **Acquired Risk for Cancer Incidence, ARCI. **A manuscript being published in the Journal of Carcinogenesis in November 2005 presents an interesting work that supports the concept of ARCI. (Cancer Incidence in the South Asian population of California, 1988–2000 Ratnali V Jain, Paul K Mills and Arti Parikh-Patel). Investigations into new paradigms like ARCI will have a far-reaching impact on the field of carcinogenesis and will open up new horizons for the scientific endeavors that seek to unravel the molecular secrets of malignant transformation of a normal cell. Several interesting questions are currently being addressed in the field of carcinogenesis research and they are expected to lead to insightful answers. Carcinogenesis Foundation and the Journal of Carcinogenesis will have tremendous opportunity to contribute to understanding the carcinogenesis processes and thereby to chemoprevention efforts.

While research is underway in several laboratories around the world, the public is waiting with a hope; a hope that a clear message that they could trust would emerge as to which factors increase the risk of carcinogenesis and which do not as the enlightened scientific community may have already deliberated and researched on them. How difficult is it to answer if vitamin E and B are protective against carcinogenesis or if they promote carcinogenesis? How difficult is it to find out if aspirin or other non-steroidal anti-inflammatory drugs prevent cancers? It may not be difficult if proper scientific methods and approaches are employed and the outcome is not prejudiced one way or the other for any reasons. It may not be difficult if socially conscious scientists approach the questions collectively with a humane outlook. As scientific approach is expected to yield one answer to one question and that answer should be verifiable in any part of the world, where is the source of discordant results to questions like those mentioned above? There seems to be two sources, one is study design and the investigators and the other is inherent in the biology of human subjects and experimental systems used for the study.

The biotechnology industry analysts seem to think that next decade will be dominated by products and processes that address the 'personalized medicine'. It certainly is the ultimate alternative to the current 'one pill cures all' approach. However, it is inevitable to go through community-based medicine before customized medicine can become a reality. **Acquired Risk for Cancer Incidence **is a concept that entails us to the community based carcinogenesis and cancer prevention research. Carcinogenesis Foundation proposes to participate in and encourage efforts that attempt to understand how the inherent biological and genetic variations in individuals influence the outcome of cancer prevention trials or cancer treatment.

While discordant results in biomedical research may have genesis in genetic diversities of subjects, a distinct contribution for this comes from lack of uniform protocols and procedures and lack of policies to prevent irresponsible scientific practices. Unfortunately, there are no international mechanisms to impose sanctions on ill trained scientists whose results misguide the public as well as the fellow scientists. It is interesting that scientific profession is the only profession that does not have certification and professional regulations like medical profession and others. It is time to think of ways and means by which a unified approach and common protocols are employed in biomedical research. It is also imperative that the individuals who go to the bench are screened and certified for technical competence and scientific integrity. These considerations may call for the creation of a body of a 'jury scientists'. Should cancer researchers be the in the forefront of such an experiment?

